# Vitamin D supplementation and prevention of cardiovascular disease and cancer in the Finnish Vitamin D Trial: a randomized controlled trial

**DOI:** 10.1093/ajcn/nqab419

**Published:** 2022-01-04

**Authors:** Jyrki K Virtanen, Tarja Nurmi, Antti Aro, Elizabeth R Bertone-Johnson, Elina Hyppönen, Heikki Kröger, Christel Lamberg-Allardt, JoAnn E Manson, Jaakko Mursu, Pekka Mäntyselkä, Sakari Suominen, Matti Uusitupa, Ari Voutilainen, Tomi-Pekka Tuomainen, Sari Hantunen

**Affiliations:** Institute of Public Health and Clinical Nutrition, University of Eastern Finland, Kuopio, Finland; Institute of Public Health and Clinical Nutrition, University of Eastern Finland, Kuopio, Finland; Independent scientist, Kangasala, Finland; Department of Biostatistics, School of Public Health and Health Sciences, University of Massachusetts, Amherst, MA, USA; Department of Epidemiology and Health Promotion and Policy, School of Public Health and Health Sciences, University of Massachusetts, Amherst, MA, USA; Australian Centre for Precision Health, Unit of Clinical and Health Sciences, University of South Australia, Adelaide, Australia; South Australian Health and Medical Research Institute, Adelaide, Australia; Institute of Public Health and Clinical Nutrition, University of Eastern Finland, Kuopio, Finland; Department of Orthopaedics, Traumatology and Hand Surgery, Kuopio University Hospital, Kuopio, Finland; Department of Food and Nutrition, University of Helsinki, Helsinki, Finland; Department of Medicine, Brigham and Women's Hospital Harvard Medical School, Boston, MA, USA; Department of Epidemiology, Harvard T.H. Chan School of Public Health, Boston, MA, USA; Institute of Public Health and Clinical Nutrition, University of Eastern Finland, Kuopio, Finland; Institute of Public Health and Clinical Nutrition, University of Eastern Finland, Kuopio, Finland; Primary Health Care Unit, Kuopio University Hospital, Kuopio, Finland; Department of Public Health, University of Turku, Turku University Hospital, Turku, Finland; School of Health Sciences, University of Skövde, Skövde, Sweden; Institute of Public Health and Clinical Nutrition, University of Eastern Finland, Kuopio, Finland; Institute of Public Health and Clinical Nutrition, University of Eastern Finland, Kuopio, Finland; Institute of Public Health and Clinical Nutrition, University of Eastern Finland, Kuopio, Finland; Institute of Public Health and Clinical Nutrition, University of Eastern Finland, Kuopio, Finland

**Keywords:** vitamin D, supplementation study, randomized controlled trial, elderly, cardiovascular disease, cancer

## Abstract

**Background:**

Vitamin D insufficiency is associated with risks of cardiovascular diseases (CVD) and cancer in observational studies, but evidence for benefits with vitamin D supplementation is limited.

**Objectives:**

To investigate the effects of vitamin D_3_ supplementation on CVD and cancer incidences.

**Methods:**

The study was a 5-year, randomized, placebo-controlled trial among 2495 male participants ≥60 years and post-menopausal female participants ≥65 years from a general Finnish population who were free of prior CVD or cancer. The study had 3 arms: placebo, 1600 IU/day, or 3200 IU/day vitamin D_3_. Follow-up was by annual study questionnaires and national registry data. A representative subcohort of 551 participants had more detailed in-person investigations. The primary endpoints were incident major CVD and invasive cancer. Secondary endpoints included the individual components of the primary CVD endpoint (myocardial infarction, stroke, and CVD mortality), site-specific cancers, and cancer death.

**Results:**

During the follow-up, there were 41 (4.9%), 42 (5.0%), and 36 (4.3%) major CVD events in the placebo, 1600 IU/d (compared with placebo: HR: 0.97; 95% CI: 0.63–1.49; *P* = 0.89), and 3200 IU/d (HR: 0.84; 95% CI: 0.54–1.31; *P* = 0.44) arms, respectively. Invasive cancer was diagnosed in 41 (4.9%), 48 (5.8%), and 40 (4.8%) participants in the placebo, 1600 IU/d (HR: 1.14; 95% CI: 0.75–1.72; *P* = 0.55), and 3200 IU/d (HR: 0.95; 95% CI: 0.61–1.47; *P* = 0.81) arms, respectively. There were no significant differences in the secondary endpoints or total mortality. In the subcohort, the mean baseline serum 25-hydroxyvitamin D concentration was 75 nmol/L (SD, 18 nmol/L). After 12 months, the concentrations were 73 nmol/L (SD, 18 nmol/L), 100 nmol/L (SD, 21 nmol/L), and 120 nmol/L (SD, 22 nmol/L) in the placebo, 1600 IU/d, and 3200 IU/d arms, respectively.

**Conclusions:**

Vitamin D_3_ supplementation did not lower the incidences of major CVD events or invasive cancer among older adults, possibly due to sufficient vitamin D status in most participants at baseline.

## Graphical Abstract

**Graphical Abstract:**
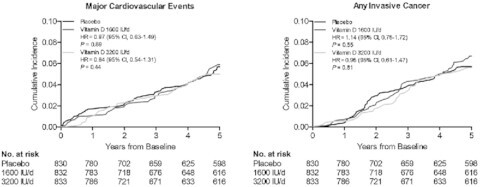

See corresponding editorial on page 1255.

## Introduction

Vitamin D, either obtained from diet or supplements or produced in the skin, is hydroxylated in the liver to 25-hydroxyvitamin D [25(OH)D], a generally used indicator of vitamin D status. Further, 25(OH)D is metabolized to the physiologically active form 1,25(OH)_2_D, which is the high-affinity ligand of the transcription factor vitamin D receptor (VDR). Genome-wide, there are more than known 10,000 VDR binding sites and significant changes in the transcriptome profile in over 1000 genes (1), suggesting a universal action of vitamin D in most tissues.

Findings from observational studies show that vitamin D insufficiency is associated with higher risks of nearly all diseases (2). At the time the Finnish Vitamin D Trial (FIND) was designed (in 2010), such observational data were already available, and ecological studies and laboratory investigations also supported a role for vitamin D in the etiology of cardiovascular diseases (CVD) and cancer (3, 4). Vitamin D was suggested to have a beneficial effect on cancer cell proliferation, apoptosis and cell differentiation, angiogenesis and metastasis, inflammation, vascular smooth muscle proliferation, blood pressure, and glucose homeostasis (3–5). However, there was little evidence from randomized controlled trials (RCT) on the potential of vitamin D supplementation to reduce the incidence of diseases. The largest RCT, with over 35,000 women, the Women's Health Initiative, tested calcium plus vitamin D_3_ (400 IU/d) supplementation for a mean of 7 years and failed to show effects on the CVD or cancer incidence (6). In another trial with 2686 older adults, 100,000 IU of vitamin D_3_ every 4 months for 5 years did not reduce the CVD or cancer incidence (7). However, in 2007, a meta-analysis of 18 RCTs summarized the effects of vitamin D supplementation and found a modestly lower risk for all-cause mortality (8). In addition, a 4-year RCT among 1179 postmenopausal female participants found that those in the calcium plus vitamin D_3_ (1100 IU/day) group were less likely to develop cancer (9). However, none of the RCTs were primarily designed to investigate the nonskeletal effects of vitamin D supplementation and in most trials the vitamin D dose was too low to induce significant changes in serum 25(OH)D concentrations. To fill this gap, large trials such as the Vitamin D and Omega-3 Trial (VITAL) (10) and the Vitamin D Assessment (ViDA) study (11, 12) were started in the early 2010s, with the aim of investigating the effects of high-dose vitamin D_3_ supplementation on CVD and cancer prevention (**Supplemental Table 1**).

Data from primary-prevention RCTs is especially scarce in the northern latitudes, like in Finland, where vitamin D production in the skin is limited to only a few summer months. According to our own research, the mean serum 25(OH)D concentration was <50 nmol/L (20 ng/mL) for most of the year in a Finnish study population (13). Lower concentrations were associated with higher risks of total and CVD mortality and with impaired glucose metabolism (13, 14). Therefore, an interventional study to investigate the impacts of high-dose vitamin D_3_ supplementation on incidences of diseases in a general aging Finnish population was highly warranted. As a unique feature among the large vitamin D_3_ supplementation trials, an aim was to also investigate dose-responses with 2 different vitamin D_3_ doses.

## Methods

### Trial design

FIND was a 5-year, randomized, double-blind, placebo-controlled supplementation trial of the effects of vitamin D_3_ on the incidences of CVD and cancer among generally healthy, White, male participants ≥60 years and postmenopausal female participants (≥65 years) from the general Finnish population, conducted in 2012–2018. The inclusion criteria were: male participants aged ≥60 years and postmenopausal female participants aged ≥65 years without a history of cancer (except nonmelanoma skin cancer) or CVD (including myocardial infarction, stroke, transient ischemic attack, angina pectoris, coronary artery bypass grafting, or percutaneous coronary intervention). The exclusion criteria were: *1*) a history of kidney stones, renal failure or dialysis, hypercalcemia, hypo- or hyperparathyroidism, severe liver disease (cirrhosis), or sarcoidosis or other granulomatous diseases, such as active chronic tuberculosis or Wegener's granulomatosis; and *2*) use of vitamin D >800 IU/d or calcium >1200 mg/d from all supplemental sources combined, unless the participant was willing to decrease or forego such use during the trial.

**FIGURE 1:**
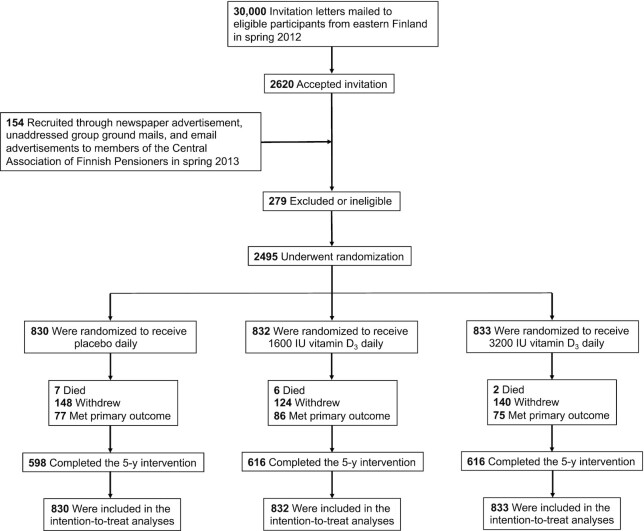
Recruitment and randomization of the study participants.

The original goal was to recruit 18,000 participants, with 3000 male and 3000 female participants in each arm. Unfortunately, the interest of the aging, generally healthy population to participate in the study turned out to be far lower than anticipated. With data obtained from the Finnish Population Register Center, 30,000 invitation letters were mailed to men aged ≥60 years and women aged ≥60 years who were living in Eastern Finland in spring 2012 (**Figure 1**). There were 2620 participants who accepted the invitation (8.7% response rate). Subsequent recruitment strategies included newspaper advertisements (200,000 copies distribution), unaddressed group ground mails (to 100,000 households), and email advertisements (80,000 members of The Central Association of Finnish Pensioners). These strategies yielded only 154 responses combined, so the recruitment was stopped in spring 2013 and the study was continued with 2495 eligible participants. However, as shown in our earlier reports (13, 14), even a moderate number of study participants may produce statistically significant differences in hard endpoints. Further, in our earlier interventional study with a similar supplementation protocol as in the current study, the achieved difference in the serum 25(OH)D concentrations between the placebo and the 3200 IU/d vitamin D_3_ arms at the end of the study was 40 nmol/L (16 ng/mL) (15), which was of the same magnitude as the difference between the extreme 25(OH)D categories in our observational study (13).

The 2495 participants were randomized to receive either *1*) 1600 IU/day of vitamin D_3_; *2*) 3200 IU/day of vitamin D_3_; or *3*) placebo. Gender-stratified simple randomization in a 1:1:1 ratio was carried out by a statistician who was not a member of the FIND study group, based on computerized random number generation. Randomization was done separately for the larger mail-only cohort and for the subcohort.

The intervention period ran between September 2012 and October 2018. A random subcohort of 600 participants (100 male and 100 female participants from each arm) was invited for more detailed examinations to the University of Eastern Finland's Kuopio campus (latitude 63°N). In total, 307 male and 244 female volunteers took part in the baseline examinations, with slightly lower numbers participating in the follow-up visits after 6, 12, and 24 months **(Supplemental Figure 1**).

The study pills were purchased from Galena Pharma Ltd. The pills were otherwise identical, but contained either 0, 1600 IU, or 3200 IU of vitamin D_3_ per pill. The supplements were in identical containers, with group codes not revealing the intervention printed on the label. Annually, the containers were either mailed to the participants or given at study visits. A double-blind setting was maintained throughout the study.

The participants filled out questionnaires at baseline; after 12, 24, and 36 months; and at the trial's end at 60 months. The questionnaires included detailed questions about sun exposure habits, physical activity, smoking, medication and supplementation use, diseases, fractures and falls, coherence, sleep, mood, mental health, functional capacity, pain, and potential side effects of the supplements during the previous 12 months. BMI was calculated based on the weight and height reported in the baseline questionnaire. At baseline, at 36 months, and at the trial's end, the questionnaire also included a validated 142-item FFQ. The final questionnaire also included a question of adherence: “how much of the study pills did you take during the study?” The choices ranged from “<50%” to “100% or almost 100%.” The participants could choose to complete the questionnaires online or could use a prepaid envelope to mail the questionnaire to the study center.

Serum 25(OH)D_3_ concentrations were measured using high-performance liquid chromatography from the samples collected at the baseline and after 6 and 12 months, complemented with direct competitive chemiluminescence immunoassays for samples collected at 5 time points (**Supplemental Methods 1**).

The study was carried out in accordance with The Code of Ethics of the World Medical Association (Declaration of Helsinki). All participants were volunteer adults who were entitled to withdraw from the study at any time without explanation. All participants signed a written informed consent form. The appropriate study approvals were obtained from the ethics committee of the Kuopio University Hospital (#30/2010) and from the National Institute for Health and Welfare, which administers the health registries.

### Endpoint assessment

Morbidity and mortality data were obtained by linkages to the national registries. The National Death Registry, in collaboration with Statistics Finland (license TK-53–507–20), was used for the mortality data. The Care Registers for Social Welfare and Health Care, including inpatient care (HILMO) and outpatient visits (AvoHILMO), governed by the National Institute for Health and Welfare (license THL/523/5.05.00/2020), were used to collect data on diseases diagnosed during the follow-up. Validation of CVD and cancer endpoints is described in the registries’ websites (16–18). There were no losses to follow-up.

The prespecified primary endpoints were any invasive cancer (International Classification of Diseases Tenth Edition codes C00–97, excluding nonmelanoma skin cancer C44) and a major CVD event [composite endpoint of myocardial infarction (I21–23), stroke (I60–64) and CVD mortality (I00–99)]. The secondary endpoints were *1*) an expanded composite CVD endpoint of myocardial infarction, stroke, CVD mortality, and coronary revascularization [i.e., coronary artery bypass grafting (Z95.1) or percutaneous coronary intervention (Z95.5)], and *2*) the individual components of the primary CVD endpoint, particularly total CVD mortality, *3*) incident colorectal cancer (C18–20), *4*) incident breast cancer (C50) in women, *5*) incident prostate cancer (C61) in men, and *6*) total cancer mortality (C00–97).

### Statistical Analysis

The study was designed to have >80% statistical power to detect a 25% decrease in CVD and cancer events in the high-dose supplementation arm compared to the placebo arm. The sample size calculations were based on numbers of CVD deaths and cancer events in the Finnish population ≥60 years, using data provided by Statistics Finland (19) and the Finnish Cancer Registry (20). With respect to a 5-year cumulative reduction in incidence rates, the required sample size per arm was 2572 to cover both CVD deaths and cancer events (**Supplemental Methods 2**). To be prepared for dropouts and noncompliance, the sample size was set to 6000 per arm.

A Cox proportional-hazards model was used to predict hazards of endpoints in the 3 arms. Because of the low number of events, in the post hoc analyses we also analyzed the effects after combining the 2 vitamin D arms. The validity of the proportional hazards assumption was evaluated using Schoenfeld residuals. Participants contributed follow-up time from randomization until the endpoint, death, end of the 5-year follow-up, or withdrawing from the study due to personal reasons (*n *= 412). The models were adjusted for age and sex. To detect trends in the hazards with respect to the intervention, we computed the log-rank test for trend applying the “survMisc” package (https://cran.r-project.org/web/packages/survMisc/index.html) for the R language. Statistical significance of the interactions with age, sex, and BMI were assessed by adding interaction terms in Cox regressions. Due to a low number of events, the interaction analyses were done only with the primary study endpoints of major cardiovascular event and any cancer. A *t*-test, Mann-Whitney U test, and Pearson's chi-squared test were used to test differences in baseline characteristics between the subcohort of 551 participants and the 1944 other participants. IBM SPSS Statistics version 25 and R version 3.5.1 (https://www.R-project.org/) were used for analyses. A 2-sided *P* value < 0.05 was used to determine statistical significance.

## Results

**Table 1** shows the baseline characteristics of the participants. The mean age was 68.2 years, and 43% were women. There were no major differences between the 3 arms at baseline. When compared to the other participants, the participants in the subcohort were younger, had a higher education, were more likely to be married, were more likely to use their own vitamin D supplements, and were more likely to rate their health as good or excellent (**Supplemental Table 2**). They also had more often been on vacation in a sunny place during the previous 12 months and more commonly used sunscreen in the summertime.

**TABLE 1:** Baseline characteristics of the participants

Characteristic	Overall (*n *= 2495)	Placebo arm (*n *= 830)	Vitamin D_3_ 1600 IU/d arm (*n *= 832)	Vitamin D_3_ 3200 IU/d arm (*n *= 833)
Female sex, *n* (%)	1069 (42.8)	372 (44.8)	349 (41.9)	348 (41.8)
Age, mean (SD), years	68.2 (4.5)	68.2 (4.5)	68.1 (4.5)	68.3 (4.4)
Age group, *n* (%)				
60–64 years^1^	620 (24.8)	202 (24.3)	209 (25.1)	209 (25.1)
65–69 years	1089 (43.6)	373 (44.9)	358 (43.0)	358 (43.0)
70–74 years	589 (23.6)	186 (22.4)	203 (24.4)	200 (24.0)
≥75 years	197 (7.9)	69 (8.3)	62 (7.5)	66 (7.9)
Employment status, *n* (%)	*n *= 2469	*n *= 823	*n *= 824	*n *= 822
Full-time work	201 (8.1)	76 (9.2)	61 (7.4)	64 (7.8)
Part-time work	95 (3.8)	27 (3.3)	38 (4.6)	30 (3.6)
Unemployed	61 (2.5)	14 (1.7)	25 (3.0)	22 (2.7)
Retired	2097 (84.9)	703 (85.4)	692 (84.0)	702 (85.4)
Not working for other reasons	15 (0.6)	3 (0.4)	8 (1.0)	4 (0.5)
Leisure-time physical activity,^2^ mean (SD), h/week				
Light	13.1 (10.8) (*n *= 2172)	13.1 (10.9) (*n *= 712)	13.3 (11.2) (*n *= 721)	12.8 (10.5) (*n *= 739)
Heavy	5.7 (6.5) (*n *= 1601)	5.6 (6.3) (*n *= 525)	5.8 (6.6) (*n *= 532)	5.9 (6.5) (*n *= 544)
Smoking regularly,^3^*n* (%)	885 (35.7) (*n *= 2477)	292 (35.4) (*n *= 825)	300 (36.5) (*n *= 823)	293 (35.3) (*n *= 829)
At least high school diploma, *n* (%)	417 (16.8) (*n *= 2483)	135 (16.3) (*n *= 827)	149 (18.0) (*n *= 827)	133 (16.0) (*n *= 829)
Married, *n* (%)	1849 (74.7) (*n *= 2476)	620 (75.2) (*n *= 824)	606 (73.5) (*n *= 825)	623 (75.3 (*n *= 827)
BMI, mean (SD), kg/m^2^	27.1 (4.3) (*n *= 2491)	27.2 (4.3) (*n *= 828)	27.1 (4.3) (*n *= 832)	26.9 (4.3) (*n *= 831)
Alcohol intake, mean (SD), g/day	7 (13) (*n *= 2464)	8 (16) (*n *= 824)	7 (11) (*n *= 818)	7 (11) (*n *= 822)
Vitamin D intake from diet, mean (SD), IU/d	428 (312) (*n *= 2464)	416 (260) (*n *= 824)	456 (384) (*n *= 818)	420 (280) (*n *= 822)
The major vitamin D sources in diet				
Liquid dairy products, mean (SD), g/d	488 (409)	473 (387)	518 (413)	474 (426)
Fish, mean (SD), g/d	76 (96)	71 (74)	83 (132)	72 (71)
Vegetable fat spreads, mean (SD), g/d	13 (9)	12 (9)	13 (9)	13 (9)
Use of own vitamin D supplements, *n* (%)				
Not at all	1670 (66.9)	535 (64.5)	566 (68.0)	569 (68.3)
200–400 IU/day	361 (14.5)	133 (16.0)	104 (12.5)	124 (14.9)
>400 to <800 IU/day	74 (3.0)	27 (3.3)	26 (3.1)	21 (2.5)
800 IU/day	390 (15.6)	135 (16.3)	136 (16.3)	119 (14.3)
Calcium intake from diet, mean (SD), mg/d	1369 (739) (*n *= 2463)	1344 (708) (*n *= 824)	1413 (735) (*n *= 817)	1350 (772) (*n *= 822)
Daily calcium supplement use, *n* (%)	381 (16.0) (*n *= 2387)	143 (18.1) (*n *= 792)	112 (14.1) (*n *= 792)	126 (15.7) (*n *= 803)
Daily medication use, *n* (%)	1736 (70.1) (*n *= 2476)	597 (72.3) (*n *= 826)	559 (67.9) (*n *= 823)	580 (70.1) (*n *= 827)
Hypertension medication, *n* (%)	1047 (42.3) (*n *= 2476)	353 (42.7) (*n *= 826)	344 (41.8) (*n *= 823)	350 (42.3) (*n *= 827)
Antiarrhythmic medication, *n* (%)	122 (4.9) (*n *= 2476)	45 (5.4) (*n *= 826)	48 (5.8) (*n *= 823)	29 (3.5) (*n *= 827)
Statin medication, *n* (%)	717 (29.0) (*n *= 2476)	255 (30.9) (*n *= 826)	218 (26.5) (*n *= 823)	244 (29.5) (*n *= 827)
Diabetes medication, *n* (%)	222 (9.0) (*n *= 2476)	68 (8.2) (*n *= 826)	89 (10.8) (*n *= 823)	65 (7.9) (*n *= 827)
Self-rated health good or excellent, *n* (%)	1460 (59.2) (*n *= 2467)	460 (56.0) (*n *= 822)	493 (60.3) (*n *= 818)	507 (61.3) (*n *= 827)
Vacation in a sunny place during previous 12 months, *n* (%)	663 (26.6) (*n *= 2490)	210 (25.4) (*n *= 828)	225 (27.1) (*n *= 830)	228 (n = 27.4) (*n *= 832)
Use of sunscreen in the summertime, *n* (%)	581 (23.4) (*n *= 2488)	183 (22.1) (*n *= 828)	199 (24.0) (*n *= 828)	199 (23.9) (*n *= 832)

1 All participants in this age group are men.

2 Light activity was defined as gardening and other light outdoor activities, while heavy activity was defined as physical exercise that causes sweating or heavy breathing.

3 Smoking regularly was defined as smoking almost every day during the last year.

**FIGURE 2:**
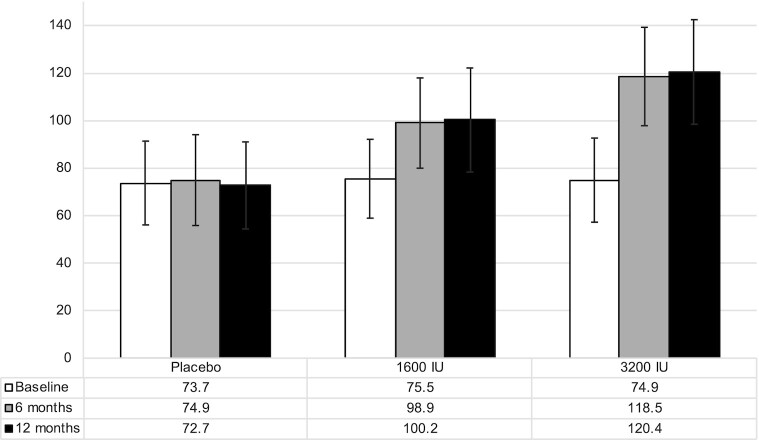
Serum 25-hydroxyvitamin D concentration at baseline and after 6 and 12 months in the placebo, 1600 IU/day and 3200 IU/day vitamin D_3_ arms among the 503 participants with data from all 3 study visits. The values are shown in nmol/L. To convert to ng/mL, divide by 2.5.

In the subcohort, the mean ± SD baseline serum 25(OH)D concentration was 74.8 ± 18.2 nmol/L (29.9 ± 7.3 ng/ml), 9.1% had concentrations <50 nmol/L (20 ng/mL), and 50.0% had concentrations ≥75 nmol/L (30 ng/mL). Among the 503 participants with complete data from the baseline, 6-month, and 12-month study visits, the mean increases in serum 25(OH)D between the baseline and 6-month visit were 23.4 nmol/l (9.4 ng/mL) in the arm receiving 1600 IU/day of vitamin D_3_ and 43.6 nmol/L (17.4 ng/mL) in the arm receiving 3200 IU/day, with little additional increase between the 6-month and 12-month visits (**Figure 2**). Few changes were observed in the placebo arm (Figure 2). Serum 25(OH)D measurements in the subgroup of 60 participants from 5 time points indicated that the differences between the arms were maintained throughout the study (**Supplemental Figure 2**). The baseline levels were lower among the male participants and those with higher BMIs, but the mean concentrations were over 90 nmol/L (36 ng/mL) in all vitamin D–supplemented arms after 12 months (**Supplemental Figure 3**).

Among the 1609 participants who completed the last questionnaire, 74.8% reported using all study pills during the study and 95.3% reported using ≥80% of the pills. The proportions were similar in the 3 arms (e.g., 95.0%, 95.7%, and 95.1% reported using ≥80% of the pills in the placebo, 1600 IU/day, and 3200 IU/day arms, respectively; *P* = 0.97).

The proportion of users of the maximum allowed personal vitamin D dose (800 IU/day) increased in all arms during the trial, but most participants reported using no personal vitamin D supplements (**Supplemental Figure 4**).

### Cardiovascular disease

**FIGURE 3:**
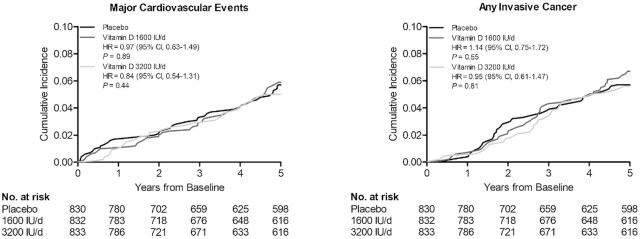
Cumulative incidence rates of major cardiovascular events and invasive cancer of any type, according to year of follow-up, in the placebo arm, 1600 IU/day vitamin D_3_ arm, and 3200 IU/day vitamin D_3_ arm. HR values are from Cox proportional hazards regression models adjusted for age and sex.

During the mean follow-up of 4.3 years (SD, 1.4 years; median, 5.0 years; minimum, 22 days; maximum, 5.0 years), 119 participants were diagnosed with a major cardiovascular event. There were no statistically significant differences in the event rates between the placebo arm and the 2 vitamin D arms (**Table 2**; **Figure 3**). There were no statistically significant differences in the event rates of the secondary outcomes of myocardial infarction, stroke, or CVD death or in the major cardiovascular events after excluding the first 2 years of follow-up (Table 2). In the analyses stratified by age, sex, and BMI, vitamin D supplementation seemed to increase the incidence of major cardiovascular events among those with lower BMIs and decrease the incidence among those with higher BMIs (**Table 3**). Although the nominal *P* value for interaction was statistically significant also for the analyses with sex, the difference in the HRs was observed only in the 1600 IU/day vitamin D group, with a higher event rate in women and a lower event rate in men (Table 3). Age did not modify the associations (Table 3).

**TABLE 2:** Primary and secondary endpoints according to randomization arm^1^

Event	Placebo (*n* = 830)	1600 IU/day (*n* = 832)	*P* value	3200 IU/day (*n* = 833)	*P* value	*P* value for trend	Combined Vitamin D Arms vs. Placebo	*P* value
PY, *n*	3480.0	3558.2		3538.4			—	
Primary endpoint: major cardiovascular event^2^								
Events, *n*	41	42		36			—	
Rate per 100 PY (95% CI)	1.18 (0.87–1.60)	1.18 (0.87–1.59)		1.02 (0.74–1.41)			—	
HR (95% CI)	1	0.97 (0.63–1.49)	0.89	0.84 (0.54–1.31)	0.44	0.53	0.90 (0.62–1.32)	0.60
Expanded major cardiovascular event endpoint^3^								
Events, *n*	43	43		37			—	
Rate per 100 PY (95% CI)	1.24 (0.92–1.66)	1.21 (0.90–1.63)		1.05 (0.76–1.44)			—	
HR (95% CI)	1	0.95 (0.62–1.44)	0.80	0.82 (0.53–1.27)	0.37	0.46	0.88 (0.61–1.28)	0.51
Myocardial infarction								
Events, *n*	18	18		20			—	
Rate per 100 PY (95% CI)	0.52 (0.33–0.82)	0.51 (0.32–0.80)		0.57 (0.37–0.88)			—	
HR (95% CI)	1	0.92 (0.48–1.77)	0.80	1.04 (0.55–1.96)	0.92	0.78	0.98 (0.56–1.71)	0.94
Stroke								
Events, *n*	18	20		16			—	
Rate per 100 PY (95% CI)	0.52 (0.33–0.82)	0.56 (0.36–0.87)		0.45 (0.28–0.74)			—	
HR (95% CI)	1	1.08 (0.57–2.05)	0.81	0.87 (0.44–1.70)	0.68	0.70	0.97 (0.55–1.72)	0.93
Cardiovascular disease death								
Events, *n*	5	5		4			—	
Rate per 100 PY (95% CI)	0.14 (0.06–0.34)	0.14 (0.06–0.34)		0.11 (0.04–0.30)			—	
HR (95% CI)	1	0.94 (0.27–3.24)	0.92	0.76 (0.20–2.83)	0.68	0.73	0.85 (0.28–2.53)	0.77
Primary endpoint: any invasive cancer								
Events, *n*	41	48		40			—	
Rate per 100 PY (95% CI)	1.18 (0.87–1.60)	1.35 (1.02–1.79)		1.13 (0.83–1.54)			—	
HR (95% CI)	1	1.14 (0.75–1.72)	0.55	0.95 (0.61–1.47)	0.81	0.85	1.04 (0.72–1.51)	0.83
Colorectal cancer								
Events, *n*	3	4		1			—	
Rate per 100 PY (95% CI)	0.09 (0.03–0.27)	0.11 (0.04–0.30)		0.03 (0.003–0.20)			—	
HR (95% CI)	1	1.28 (0.29–5.74)	0.74	0.32 (0.03–3.11)	0.33	0.37	0.80 (0.19–3.37)	0.80
Breast cancer								
Events, *n*	6	4		6			—	
Rate per 100 PY (95% CI)	0.17 (0.08–0.38)	0.11 (0.04–0.30)		0.17 (0.08–0.38)			—	
HR (95% CI)	1	0.69 (0.20–2.45)	0.57	1.08 (0.35–3.35)	0.90	0.92	0.88 (0.32–2.43)	0.81
Prostate cancer								
Events, *n*	13	11		15			—	
Rate per 100 PY (95% CI)	0.37 (0.22–0.64)	0.31 (0.17–0.56)		0.42 (0.26–0.70)			—	
HR (95% CI)	1	0.78 (0.35–1.73)	0.53	1.07 (0.51–2.24)	0.87	0.83	0.92 (0.47–1.79)	0.81
Cancer death								
Events, *n*	0	0		0			—	
Death from any cause								
Events, *n*	7	7		5			—	
Rate per 100 PY (95% CI)	0.20 (0.10–0.42)	0.20 (0.09–0.42)		0.14 (0.06–0.34)			—	
HR (95% CI)	1	0.94 (0.33–2.68)	0.91	0.68 (0.22–2.14)	0.68	0.56	0.81 (0.32–2.06)	0.66
Analyses excluding the first 2 years of follow-up								
Major cardiovascular event^2^								
Events, *n*	24	26		19			—	
Rate per 100 PY (95% CI)	0.69 (0.46–1.03)	0.73 (0.50–1.07)		0.50 (0.34–0.84)			—	
HR (95% CI)	1	1.02 (0.58–1.77)	0.95	0.76 (0.41–1.38)	0.36	0.42	0.89 (0.54–1.46)	0.64
Any invasive cancer								
Events, *n*	20	32		26			—	
Rate per 100 PY (95% CI)	0.57 (0.37–0.89)	0.90 (0.64–1.27)		0.73 (0.50–1.08)			—	
HR (95% CI)	1	1.53 (0.88–2.68)	0.13	1.26 (0.70–2.25)	0.44	0.45	1.40 (0.84–2.32)	0.33

1 Adjusted for age and sex in the Cox proportional hazards regression model. Abbreviation: PY, person-years.

2 Composite endpoint of myocardial infarction, stroke, and cardiovascular disease mortality.

3 Expanded composite endpoint of myocardial infarction, stroke, cardiovascular disease mortality, and coronary revascularization (i.e., coronary artery bypass grafting or percutaneous coronary intervention).

**TABLE 3:** HRs and 95% CIs for the primary endpoints according to subgroup and randomization arm^1^

Endpoint	Number of participants	Placebo	1600 IU/day	3200 IU/day	*P* value for trend	*P* value for interaction
Major cardiovascular event^2^						
Men (events, *n*)	1426	34	26	30		0.02
		1	0.70 (0.42–1.16)	0.81 (0.50–1.33)	0.43	
Women (events, *n*)	1069	7	16	6		
		1	2.44 (1.00–5.93)	0.91 (0.30–2.69)	0.92	
Age < median 67.4 year (events, *n*)	1247	25	16	20		0.27
		1	0.64 (0.34–1.19)	0.80 (0.45–1.45)	0.47	
Age ≥ median 67.4 year (events, *n*)	1248	16	26	16		
		1	1.49 (0.80–2.78)	0.92 (0.46–1.84)	0.87	
BMI < median 26.4 kg/m^2^ (events, *n*)	1250	9	20	23		0.003
		1	2.07 (0.94–4.55)	2.28 (1.05–4.92)	0.04	
BMI ≥ median 26.4 kg/m^2^ (events, *n*)	1245	32	22	13		
		1	0.66 (0.38–1.14)	0.41 (0.22–0.79)	0.01	
BMI <25 kg/m^2^ (events, *n*)	850	6	13	12		0.09
		1	1.93 (0.73–5.08)	1.60 (0.60–4.25)	0.39	
BMI 25–30 kg/m^2^ (events, *n*)	1136	22	24	22		
		1	1.02 (0.57–1.82)	1.01 (0.56–1.83)	0.81	
BMI ≥30 kg/m^2^ (events, *n*)	509	13	5	2		
		1	0.43 (0.15–1.21)	0.19 (0.04–0.82)	0.004	
Any cancer						
Men (events, *n*)	1426	37	38	33		0.38
		1	0.92 (0.59–1.45)	0.82 (0.51–1.31)	0.42	
Women (events, *n*)	1069	25	33	29		
		1	1.44 (0.86–2.42)	1.24 (0.73–2.12)	0.42	
Age < median 67.4 year (events, *n*)	1247	28	29	24		0.98
		1	1.02 (0.61–1.71)	0.88 (0.51–1.51)	0.63	
Age ≥ median 67.4 year (events, *n*)	1248	34	42	38		
		1	1.20 (0.76–1.89)	1.07 (0.68–1.71)	0.80	
BMI < median 26.4 kg/m^2^ (events, *n*)	1250	38	35	35		0.33
		1	0.89 (0.57–1.42)	0.84 (0.53–1.33)	0.47	
BMI ≥ median 26.4 kg/m^2^ (events, *n*)	1245	24	36	27		
		1	1.49 (0.89–2.49)	1.19 (0.69–2.07)	0.53	
BMI <25 kg/m^2^ (events, *n*)	850	24	19	26		0.39
		1	0.72 (0.39–1.31)	0.88 (0.51–1.53)	0.71	
BMI 25–30 kg/m^2^ (events, *n*)	1136	28	37	28		
		1	1.36 (0.83–2.23)	1.14 (0.67–1.93)	0.76	
BMI ≥30 kg/m^2^ (events, *n*)	509	10	15	8		
		1	1.61 (0.72–3.59)	0.91 (0.36–2.32)	0.84	

1 Adjusted for age and sex in the Cox proportional hazards regression model.

2 Composite endpoint of myocardial infarction, stroke, and cardiovascular disease mortality.

### Cancer

During the follow-up, 129 participants were diagnosed with cancer, with no statistically significant differences between the arms in the event rates in any invasive cancer or in the secondary endpoints of colorectal, breast, or prostate cancer (Table 2; Figure 3). Excluding the first 2 years of follow-up did not alter the finding for any invasive cancer (Table 2). During the follow-up, 11, 14, and 10 participants died due to cancer in the placebo, 1600 IU/day, and 3200 IU/day vitamin D_3_ arms, respectively, but these were not first incident events (Table 2). Neither age, sex, nor BMI modified the effects (Table 3).

### All-cause mortality and adverse effects

In total, 19 participants had death as the first incident event, without statistically significant differences in the event rates between the 3 arms (Table 2). There were 0, 1, and 1 cases of hypercalcemia and 7, 3, and 6 cases of urinary tract stones in the placebo, 1600 IU/day, and 3200 IU/day vitamin D_3_ arms, respectively (age- and sex-adjusted *P* values > 0.18).

## Discussion

In this population-based RCT among elderly male and female participants, vitamin D_3_ supplementation with doses of 1600 IU/day or 3200 IU/day for a mean of 4.3 years did not reduce the incidences of CVD, invasive cancer, death, or any prespecified individual CVD or cancer endpoints. Vitamin D doses also appeared safe, as the rates of adverse effects did not differ between the arms.

The lack of benefit on CVD outcomes and mortality is in line with the previous primary prevention trials. In the VITAL study, daily vitamin D_3_ supplementation did not reduce incidences of major CVD events, any of the individual CVD outcomes, or total mortality when compared to the placebo group (Supplemental Table 1) (10). A similar lack of benefit was observed with monthly vitamin D_3_ supplementation in the ViDA study (Supplemental Table 1) (11). A recent meta-analysis summarized the results from 21 trials and concluded that vitamin D supplementation does not reduce overall CVD events; individual CVD outcomes of myocardial infarction, stroke, or CVD mortality; or all-cause mortality (21). Only 4 trials had CVD as a prespecified primary study outcome, with no benefit observed (10, 11, 22, 23). Additionally, there were no significant effects in the sensitivity analyses based on, for example, sex, baseline 25(OH)D level, BMI, vitamin D dosage, daily compared with bolus dosing, concurrent use of calcium supplements, or inclusion of only postmenopausal female participants (21). Age seemed to modify the effects, as a reduced major CVD event rate was observed among older participants, but, as the authors of that study state, this finding should be interpreted cautiously because they did not control for multiple comparisons (21). In our study, sex and BMI, but not age, appeared to modify the effects of vitamin D on major CVD events. However, given the low number of events in these stratified analyses and that none of the *P* values would be considered statistically significant after correction for multiple comparisons, these findings need to be interpreted cautiously.

A recent meta-analysis reported that vitamin D supplementation did not reduce the total cancer incidence overall or in analyses stratified by several potential modifying factors (24), and our findings are in line with that observation. However, the meta-analysis did find that vitamin D supplementation decreased the incidence of cancer death (24). This was also observed in the VITAL study, which found a 25% lower risk of death from cancer in the analyses that excluded the first 2 years of follow-up (12). We were not able to investigate this outcome, because there were no cancer deaths as the first incident event. Also, the numbers of prespecified secondary cancer outcomes—colorectal, breast and prostate cancer—were very low, but the lack of an effect on these outcomes agrees with the findings in other trials (10, 25, 26).

A strength of our study was the use of 2 vitamin D doses, which enabled an investigation of dose-response. There were clear and sustained differences in the serum 25(OH)D concentrations between the arms, with the achieved mean level of 120 nmol/L (48 ng/mL) in the 3200 IU/day arm being close to the upper limit of 125–150 nmol/L (50–60 ng/mL) suggested by the US Institute of Medicine (27). Other strengths include the population-based recruitment method and the use of national health records to collect data on incident events. The extensive data collected with the study forms during the trial also allow for future investigations of several health outcomes, many of which have limited dose-response data available. Limitations include the much lower than expected recruitment rate and the lower than predicted event rates, which significantly limited the power, and the small number of participants with detailed examinations, which prevented analyses stratified by the baseline serum 25(OH)D concentrations. However, as only 9.1% of the subcohort had insufficient concentrations, we most likely would not have had enough power for these analyses in the whole population. The rather high baseline 25(OH)D concentrations most likely reflect the national food fortification policies implemented in 2003 and 2010, which increased the average vitamin D intake in the Finnish population, and the increased use of vitamin D supplements. Between 2000 and 2011, the mean vitamin D intake from diet alone increased from 140 IU/day in men and women to 560 IU/day in men and 480 IU/day in women, with parallel increases in the mean serum 25(OH)D concentrations, from 48 nmol/L to 65 nmol/L (from 19 to 26 ng/mL) (28). Because of the location of Finland in the northern hemisphere, cutaneous vitamin D production is limited to the few summer months. Because most of the participants gave blood samples outside the summer months, vitamin D production in the skin is an unlikely explanation for the high baseline 25(OH)D concentrations. The low response rate to the study invitation raises the possibility of selection bias; in other words, the vitamin D status of the FIND study population may not reflect the general elderly population in Finland. However, although comparison of the serum 25(OH)D concentrations between studies is somewhat problematic due to the lack of a standardized serum vitamin D assay method and differing methods for estimating dietary intakes, the mean serum 25(OH)D concentration and vitamin D intake in our study were generally similar to the population-level estimates (28). The 5-year duration of the study may have been too short to detect effects on incidences of CVD and cancer. However, the follow-up for incident events from the national registries will continue for at least 10 years, so in the future we can investigate potential delayed effects of vitamin D supplementation on disease incidences. Finally, as the study population consisted only of White, Finnish adults with relatively high baseline serum 25(OH)D concentrations, the results may not be generalizable to more ethnically diverse populations or to populations with higher prevalences of vitamin D insufficiency.

In conclusion, vitamin D_3_ supplementation with 1600 or 3200 IU/day for 5 years did not reduce the incidences of major CVD events, any invasive cancer, or mortality among generally healthy and mostly vitamin D–sufficient older adults in Finland. As in other similar large trials with mainly null findings, the baseline vitamin D levels have been sufficient in a large majority of the participants (10–12). Future vitamin D supplementation trials should focus on recruiting participants with low vitamin D statuses.

## Supplementary Material

nqab419_Supplemental_File

## Notes

This study was supported by funding from the Academy of Finland (#137826), University of Eastern Finland, Juho Vainio Foundation, Medicinska Understödsföreningen Liv och Hälsa, Finnish Foundation for Cardiovascular Research, Finnish Diabetes Research Foundation, and Finnish Cultural Foundation.

The funding sources had no role in the collection, analysis, and interpretation of data; preparation, review, and approval of the manuscript; or the decision to submit this manuscript for publication.

Supplemental Tables 1 and 2, Supplemental Figures 1–4, and Supplemental Methods 1 and 2 are available from the “Supplementary data” link in the online posting of the article and from the same link in the online table of contents at https://academic.oup.com/ajcn/.

Abbreviations used: CVD, cardiovascular disease; FIND, Finnish Vitamin D Trial; RCT, randomized controlled trial; VDR, vitamin D receptor; ViDA, Vitamin D Assessment; VITAL, Vitamin D and Omega-3 Trial; 25(OH)D, 25-hydroxyvitamin D.
