# Supplementary material for: Vitamin D supplementation and prevention of cardiovascular disease and cancer in the Finnish Vitamin D Trial: a randomized controlled trial
Source: Am J Clin Nutr. 2022 Jan 4;115(5):1300–10. doi: 10.1093/ajcn/nqab419 (PMC9071497; doi:10.1093/ajcn/nqab419)
Supplement: nqab419_Supplemental_File [file nqab419_supplemental_file.docx]

Vitamin D supplementation and prevention of cardiovascular disease and cancer in the Finnish Vitamin D Trial – a randomized controlled trial

Virtanen JK, et al.

**Online Supplementary Material**

| **Table of contents** |  | **Page** |
| --- | --- | --- |
| Supplementary Methods |  | 3 |
| Measurement of serum 25-hydroxyvitamin D concentration |  | 3 |
| Standardization of the serum 25-hydroxyvitamin-D measurements |  | 3 |
| Power calculations |  | 4 |
| Supplemental Table 1 | Overview of the VITAL, ViDA and FIND studies | 7 |
| Supplemental Table 2 | Baseline characteristics of the participants in the whole cohort, in the sub-cohort of 551 participants with detailed examinations and in the 1944 participants followed with annual study questionnaires | 9 |
| Supplemental Figure 1 | Number of participants in the sub-cohort study visits in the three supplementation arms and the examinations done in each visit | 11 |
| Supplemental Figure 2 | Mean serum 25-hydroxyvitamin D concentrations at baseline and after 6, 12, 24 and 52 months among 60 participants in the placebo, 1600 IU/day and 3200 IU/day vitamin D_3_ arms | 12 |
| Supplemental Figure 3 | Serum 25-hydroxyvitamin D concentration at baseline and after 12 months among 503 participants in the placebo, 1600 IU/day and 3200 IU/day vitamin D_3_ arms, according to sex, median age and body mass index | 13 |
| Supplemental Figure 4 | Reported use of own vitamin D supplements in the placebo, 1600 IU/day and 3200 IU/day vitamin D_3_ arms at baseline and after 12, 24, 36 and 60 months during the trial | 14 |
|  |  |  |
|  |  |  |

**Supplementary methods**

**Measurement of the serum 25-hydroxyvitamin D concentrations**

Among the sub-cohort of 307 male and 244 female volunteers who visited the University of Eastern Finland’s Kuopio campus at baseline and after 6 and 12 months, the serum 25-hydroxyvitamin D [25(OH)D] concentrations were measured after hexane extraction with high-performance liquid chromatography using coulometric electrode array detection, as previously described.^1^ The interassay coefficients of variation were 4.40% (at concentration of 27.73 nmol/L, SD 1.22) and 4.33% (at 93.00 nmol/L, SD 4.03).The baseline data on serum 25(OH)D was not available for 1 person in the placebo arm.

In February 2018 (at 52 months), a random sample of 120 participants were invited to provide an additional blood sample for 25(OH)D measurements, and 116 participants accepted the invitation. Of the 116 participants, 60 were from the sub-cohort (n=20 from each supplementation arm) and 54 among those who only filled the annual study questionnaires (n=18 from the placebo and 1600 IU arms and n=20 from the 3200 IU arm). The purpose of these blood samples was to assess the difference between the study arms in 25(OH)D concentrations. Among the 60 participants in the sub-cohort, we also analyzed the serum 25(OH)D concentration from blood samples collected at the 24-month study visit and reanalyzed the 25(OH)D concentrations from blood samples taken at the baseline, 6-month and 12-month study visits, in order to have a comparable analysis method for assessment of the change in 25(OH)D concentrations during the trial. In these analyses, direct competitive chemiluminescence immunoassay (LIAISON 25 OH Vitamin D Total Assay, DiaSorin, Stillwater, MN, USA) was used, with intra- and interassay coefficients of variation of 2.9% and 4.7%, respectively. The serum 25(OH)D_3_ results were standardized against National Institute of Standards and Technology (NIST) reference values.

**Standardization of the serum 25-hydroxyvitamin D measurements**

The HPLC-CEAD method for analyzing the 25(OH)D_3_ from serum samples has been applied since 2012, and the method was published in 2013.^1^ Participation to the DEQAS program was started in 2012, round 2. National Institute of Standards and Technology (NIST) values have been obtained between October 2012 and January 2016. Altogether 60 DEQAS control samples were analyzed, and for all those samples also NIST values are available.

The equation for standardization is: NIST value = ‘HPLC-CEAD’ × 0.9715 + 3.2758, r^2^ = 0.95. The standardized concentration range is 17.37 – 125.28 nmol/L for the FIND study HPLC method. This covers 98.7, 81.0 and 77.3 % of the 25OHD_3_ results at the baseline, 6 months, and 12 months, respectively.

With the HPLC-CEAD method it was possible to separate other 25(OH)D-related forms, such as 25(OH)D_2_ and epi-25(OH)D. Selectivity of the method was high, and therefore the original values were close to the NIST values.

Additionally, 25(OH)D levels were measured by direct competitive chemiluminescence immunoassay (Liaison 25 OH Vitamin D Total Assay, DiaSorin, Stillwater, MN, USA^2^), in a subset of a total of 359 samples collected at 0-, 6-, 12-, 24- and 48-month timepoints. The immunoassay measures both 25(OH)D_3_ and 25(OH)D_2_ with 100% specificity.

The immunoassay results were converted to the standardized 25(OH)D values by developing a linear regression equation with the previously obtained standardized values of 180 serum samples from 0-, 6- and 12-month timepoints and applying the equation to all 25(OH)D immunoassay results:^3^ ’standardized’ = ’immunoassay’ × 0.9042 + 19.638, r^2^ = 0.85.

**References**

1. Nurmi T, Tuomainen TP, Virtanen J, Mursu J, Voutilainen S. High-performance liquid chromatography and coulometric electrode array detector in serum 25-hydroxyvitamin D(3) and 25-hydroxyvitamin D(2) analyses. Anal Biochem 2013;435:1-9.

2. Ersfeld DL, Rao DS, Body JJ, et al. Analytical and clinical validation of the 25 OH vitamin D assay for the LIAISON automated analyzer. Clin Biochem 2004;37:867-74.

3. Cashman KD, Dowling KG, Škrabáková Z, et al. Standardizing serum 25-hydroxyvitamin D data from four Nordic population samples using the Vitamin D Standardization Program protocols: Shedding new light on vitamin D status in Nordic individuals. Scand J Clin Lab Invest 2015;75:549-61.

**Power calculations**

Power calculations for different scenarios

| Event | *n*_1_ | Incidence rate  (Placebo) | Incidence rate  (High dose) | Difference in  incidence rate | Power  1 year | Power  5 years |
| --- | --- | --- | --- | --- | --- | --- |
| CVD death | 2404^1^ | 1.83%^2^ | 1.464% | 20% | 17% | 59% |
|  |  |  | 1.3725% | 25% | 24% | 79% |
|  |  |  | 1.281% | 30% | 34% | 92% |
| CVD death | 6000 | 1.83%^2^ | 1.464% | 20% | 35% | 93% |
|  |  |  | 1.3725% | 25% | 51% | 99% |
|  |  |  | 1.281% | 30% | 68% | 100% |
|  |  |  |  |  |  |  |
| Cancer events | 2572^1^ | 1.72%^3^ | 1.376% | 20% | 17% | 60% |
|  |  |  | 1.29% | 25% | 24% | 80% |
|  |  |  | 1.204% | 30% | 34% | 93% |
| Cancer events | 6000 | 1.72%^3^ | 1.376% | 20% | 33% | 91% |
|  |  |  | 1.29% | 25% | 49% | 99% |
|  |  |  | 1.204% | 30% | 65% | 100% |

^1^Determined as the sample size for the 80% power based on the scenario of *n*_1_ = 6000.

^2^Statistics Finland

^3^Finnish Cancer Registry, Statistics Finland

In Finland in 2011, the number of people 65 years and older was 972,875, and among them 17,789 CVD deaths occurred (Statistics Finland 2011). Consequently, the incidence rate of CVD death among people 65 years and older in 2011 was 17,789 / 972,875 × 100 ≅ 1.83%.

In Finland in 2011, the incidence rates of cancer among people 60 years and older ranged from 1.102% (women 60−69 years) to 3.613% (men 80 years and older) (Finnish Cancer Registry 2011). With respect to proportions of different age groups (60−69, 70−79, 80 and older) and genders in the Finnish population in 2011 (Statistics Finland 2011) the approximate average incidence rate of cancer among people 60 years and older was 1.72%.

**Power calculations concerning cardiovascular disease (CVD) deaths with respect to a 5-year follow-up of 6000 participants**

CVD deaths in the placebo arm after five years: 110+108+106+104+102=530 i.e. 8.83%

Year 0: No. at risk = 6000.

Year 1: No. at risk = 6000 – 110 = 5890.

Year 2: No. at risk = 6000 – 110 – 108 = 5782.

Year 3: No. at risk = 6000 – 110 – 108 – 106 = 5676.

Year 4: No. at risk = 6000 – 110 – 108 – 106 – 104 = 5572.

At the end of the follow-up: No. at risk = 6000 – 110 – 108 – 106 – 104 – 102 = 5470.

CVD deaths in the high-dose supplementation arm after five years (25% decrease in the annual incidence rate): 82+81+80+79+78=400 i.e. 6.67%

Year 0: No. at risk = 6000.

Year 1: No. at risk = 6000 – 82 = 5918.

Year 2: No. at risk = 6000 – 82 – 81 = 5837.

Year 3: No. at risk = 6000 – 82 – 81 – 80 = 5757.

Year 4: No. at risk = 6000 – 82 – 81 – 80 – 79 = 5678.

At the end of the follow-up: No. at risk = 6000 – 82 – 81 – 80 – 79 – 78 = 5600.

The expected decrease of 25% in event rates was mainly based on findings in prospective observational studies from the late 2000s that had found differences in hazard ratios of around 2.0 between extreme categories of serum 25(OH)D concentration. Based on the above numbers the statistical power regarding CVD deaths with respect to the expected 25% lower annual incidence rate in the high-dose supplementation arm is 99%. The required sample size for the power of 80% is 2404 per arm (equation below).

$$n_{1}=\frac{\left[ 1.96\times\sqrt{0.0775\times0.9225\left( 1+\frac{1}{1} \right)}+0.84\times\sqrt{0.0883\times0.9117+\left( \frac{0.0667\times0.9333}{1} \right)} \right]^{2}}{{0.0216}^{2}}=2404$$

**Reference**

Rosner B. Fundamentals of Biostatistics. 7^th^ ed. Boston, MA: Brooks/Cole. 2011.

**Power calculations concerning cancer events with respect to a 5-year follow-up of 6000 participants**

Cancer events in the placebo arm after five years: 103+101+100+98+96=498 i.e.8.30%

Year 0: No. at risk = 6000.

Year 1: No. at risk = 6000 – 103 = 5897.

Year 2: No. at risk = 6000 – 103 – 101 = 5796.

Year 3: No. at risk = 6000 – 103 – 101 – 100 = 5696.

Year 4: No. at risk = 6000 – 103 – 101 – 100 – 98 = 5598.

At the end of the follow-up: No. at risk = 6000 – 103 – 101 – 100 – 98 – 96 = 5502.

Cancer events in the high-dose supplementation arm after five years (25% decrease in the annual incidence rate): 77+76+75+74+74=376 i.e. 6.27%

Year 0: No. at risk = 6000.

Year 1: No. at risk = 6000 – 77 = 5923.

Year 2: No. at risk = 6000 – 77 – 76 = 5847.

Year 3: No. at risk = 6000 – 77 – 76 – 75 =5772.

Year 4: No. at risk = 6000 – 77 – 76 – 75 – 74 = 5698.

At the end of the follow-up: No. at risk = 6000 – 77 – 76 – 75 – 74 – 74 = 5624.

Based on the above numbers the statistical power regarding cancer events with respect to the expected 25% lower annual incidence rate in the high-dose supplementation arm is 99%. The required sample size for the power of 80% is 2572 per arm (equation below.

$$n_{1}=\frac{\left[ 1.96\times\sqrt{0.0728\times0.9272\left( 1+\frac{1}{1} \right)}+0.84\times\sqrt{0.083\times0.917+\left( \frac{0.0627\times0.9373}{1} \right)} \right]^{2}}{{0.0203}^{2}}=2572$$

**Reference**

Rosner B. Fundamentals of Biostatistics. 7^th^ ed. Boston, MA: Brooks/Cole. 2011.

Supplementary Table 1 Overview of the VITAL, ViDA and FIND studies

| Study, country | Study period | Median follow-up, y | Size | Age, y | Main inclusion criteria | Vitamin D_3_ dose and frequency | Baseline serum 25(OH)D concentration, mean (SD) | Primary outcome(s) | Main findings related to CVD or cancer incidence in the vitamin D_3_ group vs. the placebo group |
| --- | --- | --- | --- | --- | --- | --- | --- | --- | --- |
| VITAL,  United States (1) | 2011-2017 | 5.3 | 25,871 | Men 50+, women 55+ | No history of cancer (except non-melanoma skin cancer) or CVD | 2000 IU/day with or without 1 g of fish oil (2X2 design) | 77 (25) nmol/L  [31 (10) ng/mL] | Major CVD event (a composite of myocardial infarction, stroke or CVD death), any invasive cancer | No effect on the major CVD event or on any of the individual CVD outcomes of the major CVD event.  No effect on any invasive cancer, on colorectal, breast or prostate cancer, or on cancer death. For cancer death after excluding first 2 y of follow-up: HR, 0.75 (95% CI 0.59-0.96) (1).  No effect on mortality. |
| ViDA,  New Zealand (2,3) | 2011-2015 | 3.3 | 5108 | 50-84 | Age 50-84 | 200,000 IU initially, 100,000 IU monthly thereafter (⁓3,330 IU/d) | 66 (23) nmol/L  [26 (9) ng/mL] | CVD (death and hospitalization) | No effect on CVD.  In post-hoc analyses: no effect on cancer incidence. |
| FIND, Finland | 2012-2018 | 5.0 | 2495 | Men 60+, women 65+ | No history of cancer (except non-melanoma skin cancer) or CVD | 1600 IU/day or 3200 IU/day | 75 (18) nmol/L  [30 (7) ng/mL] | Major CVD event (a composite of myocardial infarction, stroke or CVD death), any invasive cancer | No effect on the major CVD event or on any of the individual CVD outcomes of the major CVD event.  No effect on any invasive cancer or on colorectal, breast or prostate cancer.  No effect on mortality. |

CI, confidence interval; CVD, cardiovascular disease; FIND, Finnish Vitamin D Trial; HR, hazard ratio; ViDA, Vitamin D Assessment study; VITAL, Vitamin D and Omega-3 Trial.

**References**

1. Manson JE, Cook NR, Lee IM, et al. Vitamin D supplements and prevention of cancer and cardiovascular disease. N Engl J Med. 2019;380:33-44.

2. Scragg R, Stewart AW, Waayer D, et al. Effect of monthly high-dose vitamin D supplementation on cardiovascular disease in the Vitamin D Assessment Study: a randomized clinical trial. JAMA Cardiol. 2017;2:608-16.

3. Scragg R, Khaw KT, Toop L, et al. Monthly high-dose vitamin D supplementation and cancer risk: a post hoc analysis of the Vitamin D Assessment randomized clinical trial. JAMA Oncol. 2018;4:e182178.

Supplementary Table 2 Baseline characteristics of the participants in the whole cohort, in the sub-cohort of 551 participants with detailed examinations and in the 1944 participants followed with annual study questionnaires

| Characteristic | Overall  (n=2495) | Sub-cohort  (n=551) | Other cohort  (n=1944) | *P*-value^1^ |
| --- | --- | --- | --- | --- |
| Female sex, n (%) | 1069 (42.8) | 244 (44.3) | 825 (42.4) | 0.44 |
| Age, mean (SD), y | 68.2 (4.5) | 67.7 (4.0) | 68.3 (4.6) | 0.001 |
| Age group, n (%) |  |  |  | 0.004 |
| 60-64 yr | 620 (24.8) | 140 (25.4) | 480 (24.7) |  |
| 65-69 yr | 1089 (43.6) | 259 (47.0) | 830 (42.7) |  |
| 70-74 yr | 589 (23.6) | 128 (23.2) | 461 (23.7) |  |
| ≥75 yr | 197 (7.9) | 24 (4.4) | 173 (8.9) |  |
| Employment status, n (%) | n=2469 | n=550 | n=1919 | 0.65 |
| Full time work | 201 (8.1) | 48 (8.7) | 153 (8.0) |  |
| Part-time work | 95 (3.8) | 26 (4.7) | 69 (3.6) |  |
| Unemployed | 61 (2.5) | 11 (2.0) | 50 (2.6) |  |
| Retired | 2097 (84.9) | 462 (84.0) | 1635 (85.2) |  |
| Not working for other reasons | 15 (0.6) | 3 (0.5) | 12 (0.6) |  |
| Leisure-time physical activity, mean (SD), h/week^2^ |  |  |  |  |
| Light | 13.1 (10.8) (n=2172) | 13.0 (10.4) (n=486) | 13.1 (11.0) (n=1686) | 0.88 |
| Heavy | 5.7 (6.5) (n=1601) | 5.4 (6.0) (n=395) | 5.9 (6.6) (n=1206) | 0.17 |
| Smoking regularly,^3^ n (%) | 885 (35.7) (n=2477) | 183 (33.4) (n=548) | 702 (36.4) (n=1929) | 0.20 |
| At least high school diploma, n (%) | 417 (16.8) (n=2483) | 139 (25.3) (n=549) | 278 (14.4) (n=1934) | <0.001 |
| Married, n (%) | 1849 (74.7) (n=2476) | 454 (82.5) (n=550) | 1395 (72.4) (n=1926) | <0.001 |
| Body mass index, mean (SD), kg/m^2^ | 27.1 (4.3) (n=2491) | 27.0 (4.1) (n=550) | 27.1 (4.3) (n=1941) | 0.73 |
| Alcohol intake, mean (SD), g/day | 7 (13) (n=2464) | 8 (12) (n=550) | 7 (13) (n=1914) | 0.03 |
| Vitamin D intake from diet, mean (SD), IU/day | 428 (312) (n=2464) | 432 (288) (n=550) | 428 (320) (n=1914) | 0.85 |
| *The major vitamin D sources in diet* |  |  |  |  |
| Liquid dairy products, mean (SD), g/day | 488 (409) | 482 (379) | 490 (417) | 0.71 |
| Fish, mean (SD), g/day | 76 (96) | 73 (88) | 76 (99) | 0.55 |
| Vegetable fat spreads, mean (SD), g/day | 13 (9) | 13 (9) | 13 (9) | 0.72 |
| Use of own vitamin D supplements, n (%) |  |  |  | 0.003 |
| Not at all | 1670 (66.9) | 337 (61.2) | 1333 (68.6) |  |
| 5-10 µg/day | 361 (14.5) | 85 (15.4) | 276 (14.2) |  |
| 11-19 µg/day | 74 (3.0) | 17 (3.1) | 57 (2.9) |  |
| 20 µg/day | 390 (15.6) | 112 (20.3) | 278 (14.3) |  |
| Calcium intake from diet, mean (SD), mg/d | 1369 (739) (n=2463) | 1396 (690) (n=549) | 1361 (753) (n=1914) | 0.33 |
| Daily calcium supplement use, n (%) | 381 (16.0) (n=2387) | 98 (18.2) (n=538) | 283 (15.3) (n=1849) | 0.11 |
| Daily medication use, n (%) | 1736 (70.1) (n=2476) | 385 (70.1) (n=549) | 1351 (70.1) (n=1927) | 0.99 |
| Hypertension medication, n (%) | 1047 (42.3) (n=2476) | 237 (43.2) (n=549) | 810 (42.0) (n=1927) | 0.64 |
| Antiarrhythmic medication, n (%) | 122 (4.9) (n=2476) | 28 (5.1) (n=549) | 94 (4.9) (n=1927) | 0.83 |
| Statin medication, n (%) | 717 (29.0) (n=2476) | 162 (29.5) (n=549) | 555 (28.8) (n=1927) | 0.75 |
| Diabetes medication, n (%) | 222 (9.0) (n=2476) | 46 (8.4) (n=549) | 176 (9.1) (n=1927) | 0.59 |
| Self-rated health good or excellent, n (%) | 1460 (59.2) (n=2467) | 356 (65.4) (n=544) | 1104 (57.4) (n=1923) | 0.001 |
| Vacation in a sunny place during previous 12 months, n (%) | 663 (26.6) (n=2490) | 206 (37.6) (n=548) | 457 (23.5) (n=1942) | <0.001 |
| Use of sunscreen in summertime, n (%) | 581 (23.4) (n=2488) | 154 (27.9) | 427 (22) (n=1937) | 0.004 |

^1^Differences between groups were analyses with the Student’s t-test (continuous variables) and with Pearson’s chi^2^-test and Mann-Whitney test (categorical variables).

^2^Light activity defined as gardening and other light outdoor activities etc., heavy activity defined as physical exercise that causes sweating or heavy breathing.

^3^Smoking regularly defined as smoking almost every day during the last year.


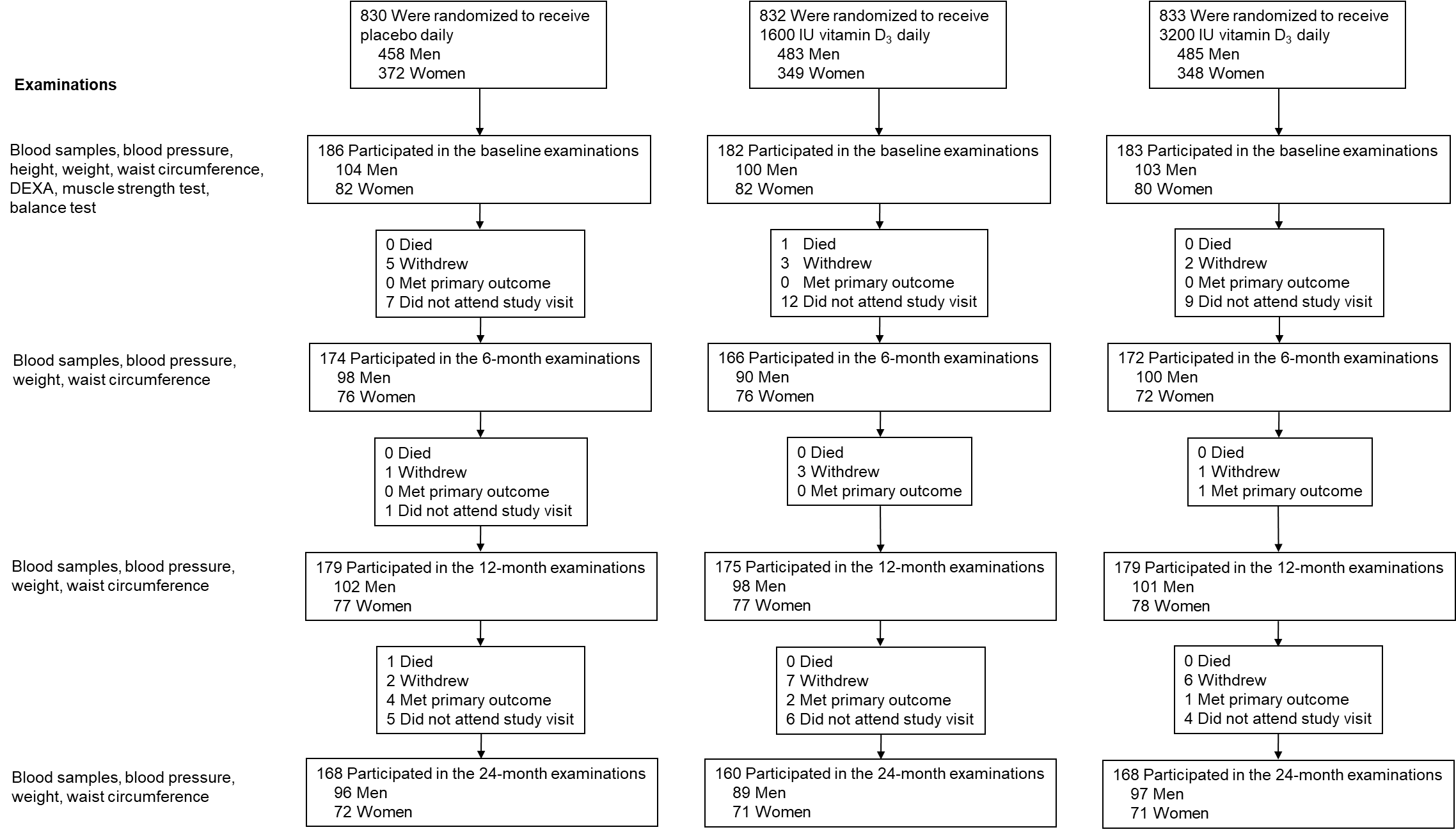


**Supplementary Figure 1. Number of participants in the sub-cohort study visits in the three supplementation arms and the examinations done in each visit.**

DEXA, Dual-energy X-ray absorptiometry.


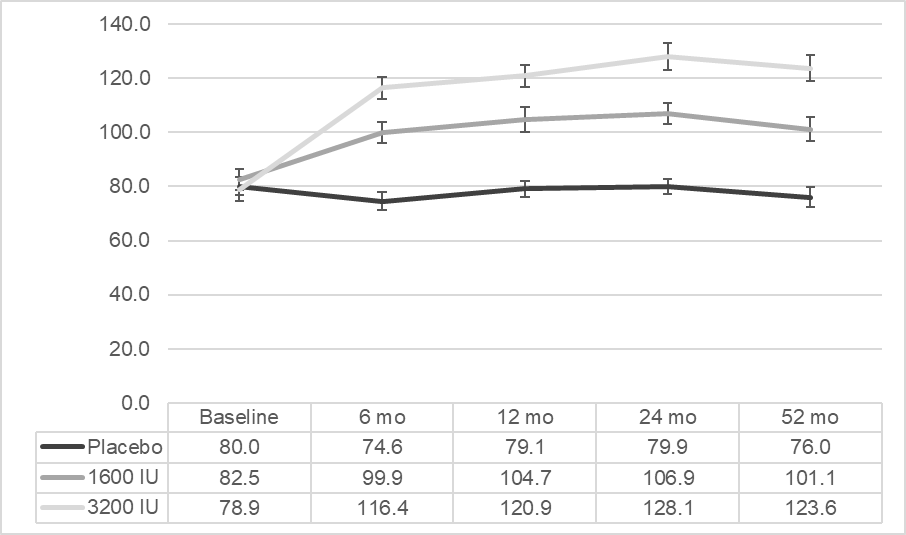


•

•

•

**Supplementary Figure 2. Mean serum 25-hydroxyvitamin D concentrations at baseline and after 6, 12, 24 and 52 months among 60 participants in the placebo, 1600 IU/day and 3200 IU/day vitamin D_3_ arms.**

The values are nmol/L. To convert the values to ng/mL, divide by 2.5. The error bars show the standard error of the mean. The dots at 52 months indicate the mean serum 25-hydroxyvitamin D concentrations among those 56 participants who gave blood samples only at the last year of the study. Among them, the mean (SD) serum 25(OH)D concentrations in the placebo, 1600 IU/day and 3200 IU/day vitamin D_3_ arms were 77.9 (17.5) nmol/L, 98.7 (19.4) nmol/L and 115.8 (27.9) nmol/L, respectively (*P*<0.001).


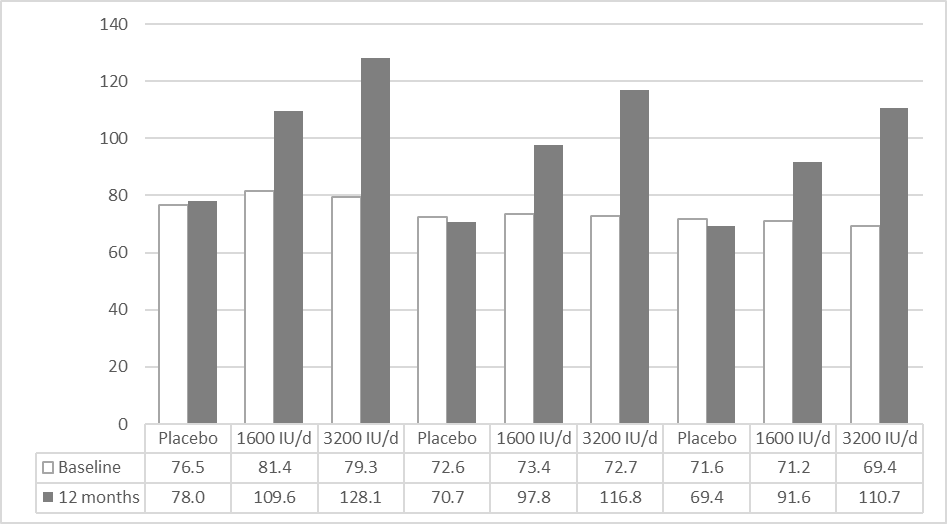

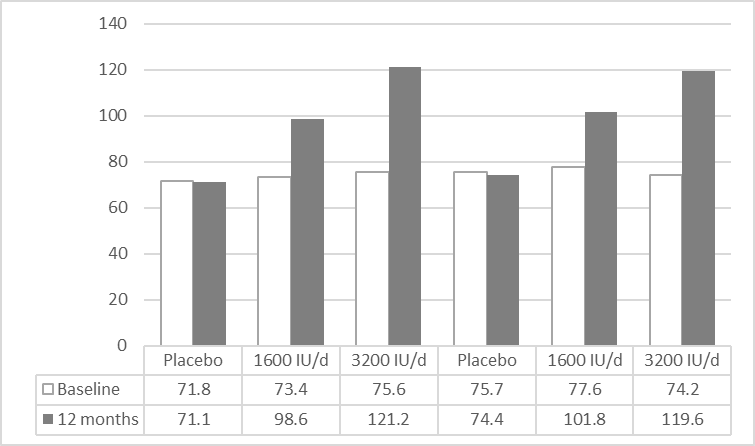

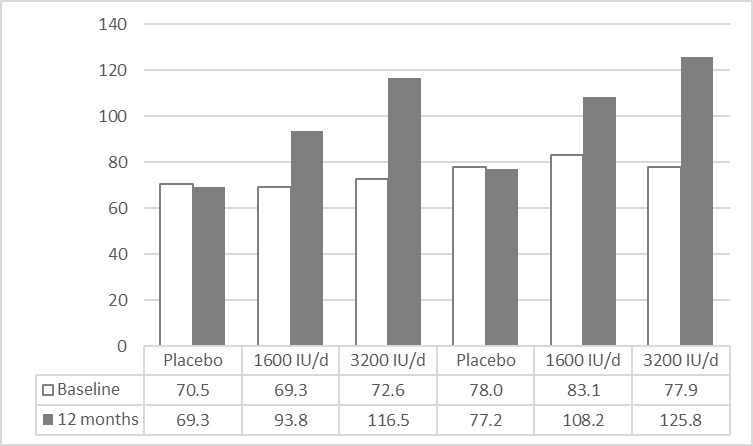


Male

Female

<67.0 yr

≥67.0 yr

25-<30 kg/m^2^

<25 kg/m^2^

≥30 kg/m^2^

**Sex**

**Body mass index**

**Age**

**Supplementary Figure 3. Serum 25-hydroxyvitamin D concentration at baseline and after 12 months among 503 participants in the placebo, 1600 IU/day and 3200 IU/day vitamin D_3_ arms, according to sex, median age and body mass index.**

The values are nmol/L. To convert to ng/mL, divide by 2.5.


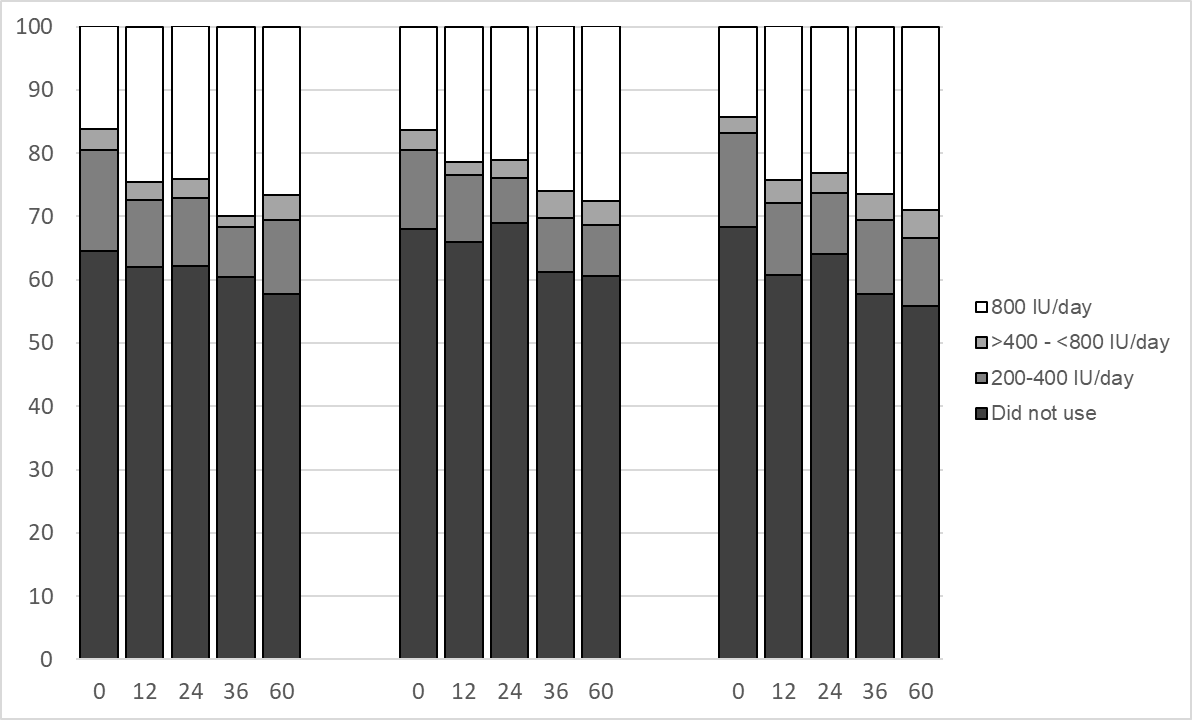


Placebo

1600 IU/day

3200 IU/day

Proportion of participants (%)

**Supplementary Figure 4. Reported use of own vitamin D supplements in the placebo, 1600 IU/day and 3200 IU/day vitamin D_3_ arms at baseline and after 12, 24, 36 and 60 months during the trial.**
